# Recent advances in retinal imaging and diagnostics

**Published:** 2019-12-17

**Authors:** Sucheta Kulkarni, Madan Deshpande

**Affiliations:** 1 Director: Clinical services and Head- Retina and ROP services, H. V. Desai Eye Hospital, Pune, India.; 2 Chief Medical Director: H. V. Desai Eye Hospital, Pune, India.

**New imaging technologies like artificial intelligence and deep learning systems show a potential to screen populations at risk of retinal diseases at a large scale in a resource constrained setting.**

**Figure 1:**
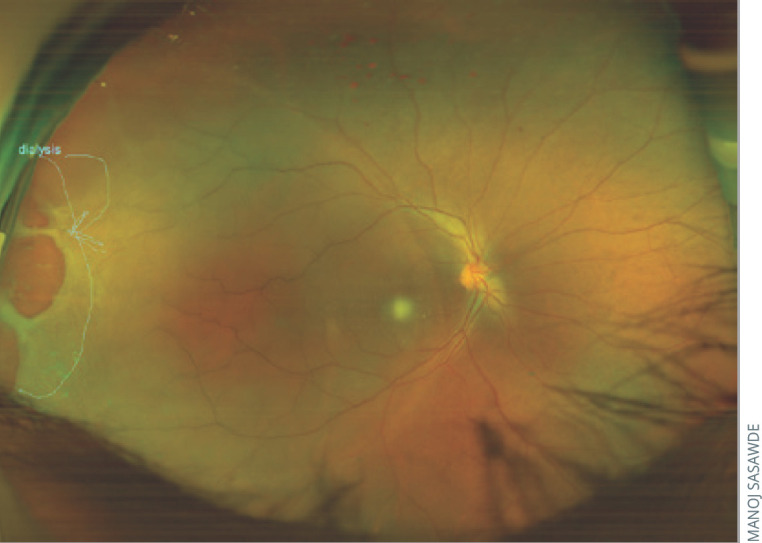
Retinal dialysis imaged with an ultra-wide field camera. INDIA

Retinal disorders are emerging as important causes of blindness in middle-income countries. In a recent rapid assessment of avoidable blindness plus diabetic retinopathy (RAAB plus DR) survey in western India, posterior segment disorders (PSD) were responsible for nearly 39 per cent of blindness next only to cataract (45 per cent).^1^ Diabetic retinopathy (DR), retinopathy of prematurity (ROP) and age-related macular degeneration (ARMD) are the important retinal diseases of public health significance.

## Challenges in screening for retinal diseases

Challenges to provision of screening in resource poor regions such as Asia include lack of specialists and lack of equipment. Of the limited specialists, most practise in urban areas, whereas a large population resides in remote rural areas. Retinal imaging devices and telemedicine can help address the ‘rural-urban gap’,^2^ as non-ophthalmologists can screen for retinal diseases to save the precious time of specialists.

## Types of retinal imaging

Several types of cameras can image the retina or optic nerve. Imaging a retina allows one to:

- screen various diseases
- photo document pathological lesions
- track disease process
- see response to therapy overtime

Fundus photography has transformed from electronic flashes to smart phone-based cameras to more recent portable eye examination kit (PEEK). PEEK is a smart phone-based application for comprehensive eye examination (www.peekvision.org). The advantage of these cameras is that a non-ophthalmologist can take pictures and, with some training, grade them as well.

We discuss various types of imaging systems below:

### Mydriatic camera

This is the most used and sophisticated imaging system available in the market. By asking patients to move their eyes in different directions, one can take images of posterior pole as well as the periphery of retina. Pupillary dilatation and bright flash lights make this system less patient friendly. The bulky size of a mydriatic camera makes it unsuitable for use in outreach/high volume screening.

### Non-mydriatic camera

Low cost and less weight makes a non-mydriatic camera ideal for screening. It also appeals to patients as you can image without pupillary dilatation and use low intensity flash light.

### Hand-held cameras

The big advantage of a hand-held camera system is its small size. This system does not need to be mounted on a table top unlike the mydriatic and non-mydriatic cameras. It's portable size and low cost make this a good option for high volume screening programmes. One important disadvantage with this camera is that, it is difficult to get good quality images if cataract or other media haze are present. Hand-held cameras cannot be used for special investigations such as FFA.

### Smart phone-based camera systems

Special adaptors make it possible to use smart phones as fundus cameras. This is the cheapest way to image a retina. In several studies, a smart phone-based retinal camera has shown similar results to a desktop fundus camera.^3^

Most cameras we mention above provide a field of view between 30 and 45 degrees. This is suitable to identify diseases that affect posterior pole like diabetic retinopathy (DR) or age-related macular degeneration (ARMD). However, there is a possibility of missing lesions in the retinal periphery.

### Ultra-wide field (UWF) camera

UWF camera helps to take images of peripheral retinal lesions like vein occlusions, vasculitis, posterior uveitis, breaks and detachments. UWF cameras provide a field of view of 200 degrees which is approximately 82 per cent of retina surface. Fundus fluorescein angiography (FFA) with such cameras helps to detect peripheral vascular lesions which are otherwise missed in a standard FFA. Figure 1 shows the ultra-wide field image of a giant retinal tear which may have missed with standard photography. Disadvantages of UWF imaging system are high cost and bulky size.

**Figure 2:**
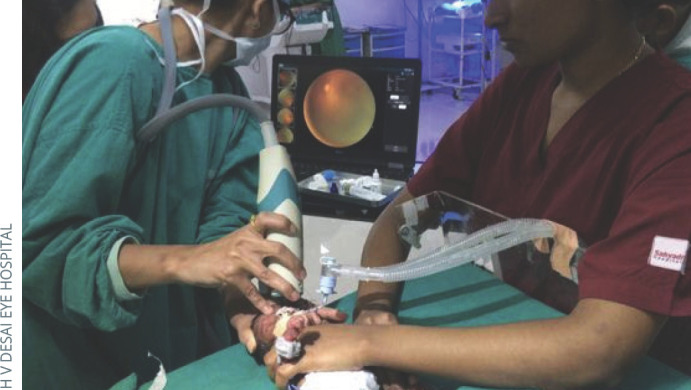
ROP screening with a portable camera with probe resting on neonate's eye. INDIA

### Ocular angiography

Angiography of the retina and choroid shows vascular diseases and inflammatory pathologies. FFA provides useful information on diabetic retinopathy, retinal vein occlusions and retinal vasculitis. For choroidal pathologies such as ARMD, the ICG angiography is a better tool. Areas where there is hypoperfusion, leakage or staining, show the anatomical location and pathological process. This helps to arrive at a conclusive diagnosis and plan for future management of a case.

### Fundus autofluorescence

Fundus autoflourescence (FAF) is non-invasive and a quick method to assess the function of retinal pigment epithelium (RPE). FAF aids in diagnosis of optic nerve head drusen, Best's disease and hereditary macular dystrophies.

### Paediatric retinal imaging

The cameras we mention above need a person to sit in front of a camera aperture and fix their gaze on a target. This is impossible in a neonate or an infant. Certain wide field imaging systems like Retcam and Forus Neo help in such cases. Some of these cameras are portable and have a probe which resembles an ultrasound transducer. Paediatric retinal imaging is a contact technique and images up to the ora serrata can be taken under topical anaesthesia. Portability makes these tools an excellent choice for screening retinopathy of prematurity (ROP) and retinoblastoma.

### Optical coherence tomography

**Figure 3:**
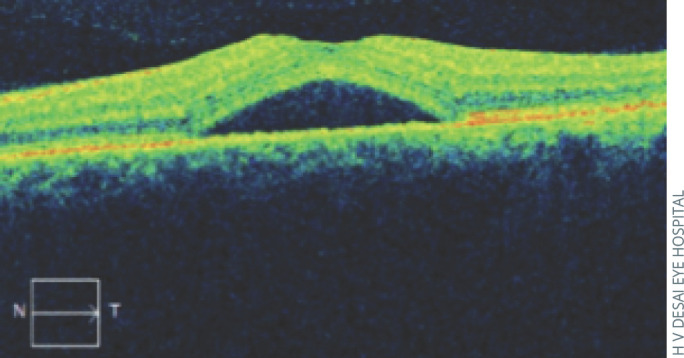
OCT scan of macula showing a neurosensory detachment. INDIA

Optical coherence tomography (OCT) is equal to a histopathological section of a tissue. One can study individual layers of retinal cells and pathological lesions in them. OCT diagnoses many subtle pathologies which may be missed during a clinical examination. Figure 3 shows an OCT image of a neurosensory detachment secondary to central serous retinopathy. OCT is performed on an undilated pupil. Newer OCT machines can capture good quality images even through dense cataracts. Recent addition of OCT angiography allows dyeless visualisation of retinal vessels in macula.

### Artificial intelligence in diagnosis of retinal conditions

Artificial intelligence (AI) and deep learning system (DLS) have the potential to improve screening coverage in resource constrained settings. In DLS, neural networks read labels of images with normal and abnormal findings. It then starts to recognise patterns and groups similar images of a particular diagnosis. More the number of image sets, higher the precision and accuracy.

In a study done in Singapore, researchers used AI and DLS to screen and identify DR and other eye diseases. The results of the study showed a very high sensitivity to vision threatening DR but a low sensitivity for diabetic macular oedema. Which makes it, one of the major limitations in a DR screening programme. However the study showed a high sensitivity and specificity for detection of glaucoma and ARMD.^4^ Further research may establish the validity of DLS in making a difference at a large scale. AI and DLS show the potential in screening programmes. They can reduce the burden on trained human resources and enable specialists to focus on treatment of these conditions.

## Conclusion

Advances in retinal imaging have led to a paradigm change in diagnosis and management of retinal diseases. In future, use of new technologies like AI and DLS in screening programmes is likely to help identify several blinding retinal conditions and treat them at an early stage.
